# The Potential Neurological Impact of Intraoperative Hyponatremia Using Histidine–Tryptophan–Ketoglutarate Cardioplegia Infusion in Adult Cardiac Surgery

**DOI:** 10.3390/medicina60060995

**Published:** 2024-06-18

**Authors:** Yu-Ning Hu, Tsung-Hao Hsieh, Sheng-Fu Liang, Meng-Ta Tsai, Chung-Yao Chien, Chung-Dann Kan, Jun-Neng Roan

**Affiliations:** 1 Division of Cardiovascular Surgery, Department of Surgery, National Cheng Kung University Hospital, College of Medicine, National Cheng Kung University, Tainan 701, Taiwan; windermere0209@gmail.com (Y.-N.H.); dongsar@gmail.com (M.-T.T.); kcd56@mail.ncku.edu.tw (C.-D.K.); 2 Department of Computer Science and Information Engineering, National Cheng Kung University, Tainan 701, Taiwan; tsung.hao.hsieh@gmail.com (T.-H.H.); sfliang@ncku.edu.tw (S.-F.L.); 3 Department of Psychology, National Cheng Kung University, Tainan 701, Taiwan; 4 Department of Neurology, National Cheng Kung University Hospital, College of Medicine, National Cheng Kung University, Tainan 701, Taiwan; ccy810826@yahoo.com.tw

**Keywords:** histidine–tryptophan–ketoglutarate solution, cardioplegia, hyponatremia

## Abstract

*Background and Objectives*: The relationship between histidine–tryptophan–ketoglutarate (HTK)-induced hyponatremia and brain injury in adult cardiac surgery patients is unclear. This study analyzed postoperative neurological outcomes after intraoperative HTK cardioplegia infusion. *Materials and Methods*: A prospective cohort study was conducted on 60 adult patients who underwent cardiac surgery with cardiopulmonary bypass. Of these patients, 13 and 47 received HTK infusion and conventional hyperkalemic cardioplegia, respectively. The patients’ baseline characteristics, intraoperative data, brain injury markers, Mini-Mental State Examination (MMSE) scores, and quantitative electroencephalography (qEEG) data were collected. Electrolyte changes during cardiopulmonary bypass, the degree of hyponatremia, and any associated brain insults were evaluated. *Results*: The HTK group presented with acute hyponatremia during cardiopulmonary bypass, which was intraoperatively corrected through ultrafiltration and normal saline administration. Postoperative sodium levels were higher in the HTK group than in the conventional cardioplegia group. The change in neuron-specific enolase levels after cardiopulmonary bypass was significantly higher in the HTK group (*p* = 0.043). The changes showed no significant differences using case–control matching. qEEG analysis revealed a significant increase in relative delta power in the HTK group on postoperative day (POD) 7 (*p* = 0.018); however, no significant changes were noted on POD 60. The MMSE scores were not significantly different between the two groups on POD 7 and POD 60. *Conclusions*: HTK-induced acute hyponatremia and rapid correction with normal saline during adult cardiac surgeries were associated with a potential short-term but not long-term neurological impact. Further studies are required to determine the necessity of correction for HTK-induced hyponatremia.

## 1. Introduction

Myocardial protection during cardiac surgery involves the use of cardioplegic solutions to minimize energy consumption and mitigate the effects of myocardial ischemia [1]. In comparison to conventional hyperkalemic blood cardioplegic solutions, the histidine–tryptophan–ketoglutarate (HTK) solution provides a longer duration of myocardial protection and eliminates the need for repeated administration [2]. Both HTK and conventional cardioplegic solutions have been approved for use in myocardial protection by the U.S. Food and Drug Administration [3]. The cardioplegic mechanism of HTK involves intracellular hyperpolarization, which is facilitated by its low sodium and potassium contents [4]. HTK cardioplegia has demonstrated excellent efficacy in myocardial protection and is used in adult and pediatric patients [4,5,6,7,8]. However, the administration of HTK solution during myocardial ischemia can lead to acute hyponatremia owing to its low sodium content [9], potentially resulting in cerebral swelling [10]. Electroencephalography (EEG) findings in hyponatremia cases typically exhibit nonspecific EEG slowing with severe hyponatremia, initially causing posterior slowing followed by diffuse delta activity [11].

The use of HTK solution during cardiopulmonary bypass (CPB) can induce hyponatremia, which may be associated with postoperative seizures in pediatric patients [9,12]. The occurrence of acute hyponatremia during HTK cardioplegia has been observed in adults; however, hyponatremia correction is not always recommended because of the potential risk of hypertonicity and subsequent adverse events [13]. Nevertheless, the potential brain insults caused by fluctuating sodium levels after HTK solution administration in adult patients remain elusive.

This study investigated the potential occurrence of neurological insults associated with perioperative acute hyponatremia after HTK infusion in adult patients.

## 2. Materials and Methods

**Figure 1:**
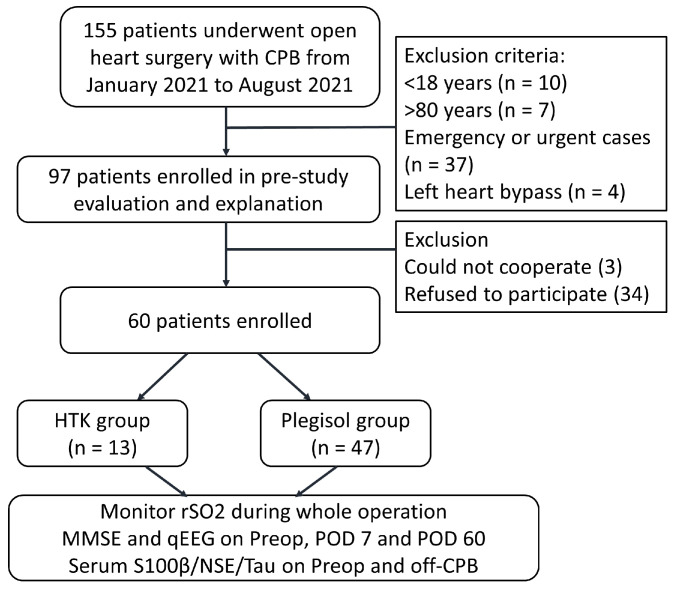
Flowchart showing enrollment of patients. Abbreviations: CPB, cardiopulmonary bypass; MMSE, Mini-Mental State Examination; NSE, neuron-specific enolase; Preop, preoperative; POD 7, postoperative day 7; POD 60, postoperative day 60; rSO_2_, regional cerebral oxygen saturation.

This study used a prospective cohort design, as previously described [14]. A total of 60 patients were enrolled in the study. Among them, 13 patients received the HTK solution (HTK group), whereas the remaining 47 patients received the Plegisol solution (Plegisol group) during cardiac surgeries (Figure 1). The study protocol was approved by the institutional review board (NCKUH IRB: B-BR-109-092-T and A-ER-110-299). Written informed consent was obtained from all participants, as evidenced by their signed documentation. Between January 2021 and August 2021, adult patients who underwent cardiac surgery with CPB at NCKUH were prospectively enrolled. Patients scheduled to undergo coronary artery bypass grafting, valve surgery, aortic surgery, and other forms of cardiac surgery were included in the study. The exclusion criteria encompassed patients scheduled for descending aorta surgery with left heart bypass or emergency surgery, and those who exhibited either a critical preoperative condition or an extremely impaired level of consciousness, rendering them unsuitable for Mini-Mental State Examination (MMSE).

The choice between HTK (Custodiol^®^ HTK) or Plegisol (Plegisol^®^ Pfizer) cardioplegia was made at the discretion of the surgeons. At our institution, HTK is not preferred during isolated coronary artery bypass grafting surgeries because of the relatively short ischemic time. In adult patients, right atriotomy is not performed to extract the HTK solution from the coronary sinus. Hence, the solution is returned to the CPB circuit. Acute hyponatremia resulting from HTK cardioplegia is managed by prompt correction using ultrafiltration with normal saline (0.9% sodium chloride) during CPB, with a target sodium level of >135 mmol/L before weaning off CPB.

Preoperative and postoperative assessments were performed as described previously [14]. In brief, MMSE and quantitative EEG (qEEG) were conducted 1 day before surgery, 1 week postoperatively (POD 7), and 2 months postoperatively (POD 60). Serum brain injury markers, including S100β, neuron-specific enolase (NSE), and Tau protein, were sampled 1 day before surgery and at completion of CPB. Baseline; time at initiating CPB; post-cross-clamp (XC); off-XC; and postoperative day 1 (POD 1) electrolyte, glucose, osmolality, and Hct data were recorded. Clinical outcomes include postoperative neurological deficits as the primary outcome. Other secondary outcomes include postoperative changes in brain injury markers, MMSE scores, qEEG, and other adverse clinical events.

### 2.1. qEEG Analysis

The qEEG evaluation, as described previously [14], was performed in a quiet environment with the patient lying on a tilted bed, adjustable to an inclination of 45°–60° for optimal comfort. During EEG recording, patients were instructed to close their eyes and relax but remain alert for 5–10 min. Alertness was monitored by evaluating the EEG signals and confirming the patient’s alertness during recording. Each patient underwent at least two 5-minute sessions of resting-state EEG.

Scalp EEG was continuously recorded in DC mode at a sampling rate of 1000 Hz using eight electrodes (T7, F3, C3, P3, F4, C4, P4, and T8) following the 10–20 international system. All scalp electrodes were referenced to the linked left ear bone, with the right ear bone serving as the ground. The electrode impedance was maintained at ≤5 KΩ. The EEG signals were amplified using the NuAmps Neuroscan device (Compumedics, Charlotte, NC, USA) and filtered online with a low-pass filter (DC to 100 Hz, 6-dB/octave attenuation).

Offline EEG analysis was conducted using the EEGLAB toolkit (http://sccn.ucsd.edu/eeglab, accessed on 13 January 2022, (Delorme and Makeig, 2004)) [15] in the MATLAB environment (Version 2020b, The MathWorks, Natick, MA, USA). Each EEG recording was edited to remove the first and last 5 s to avoid artifacts in the experimental setup. The EEG data were then band-pass-filtered between 0.5 and 50 Hz and segmented into 5 s epochs. These epochs were inspected for large eye, muscle, or other movement artifacts (maximum amplitude or power spectra of >100 µV/40 dB) and removed as necessary.

After merging all recording sessions, patients with >2 min (24 epochs) of usable data were included in the spectrum analysis. For each patient, the average power spectra were calculated using fast Fourier transforms at each electrode. The power was analyzed in the following frequency bands: delta (1–4 Hz), theta (5–7 Hz), alpha (8–12 Hz), low beta (13–16 Hz), mid-beta (17–20 Hz), high beta (21–28 Hz), all beta (13–28 Hz), and low gamma (30–50 Hz).

### 2.2. Statistical Analysis

Continuous variables are expressed as median and 25th–75th percentile, whereas categorical variables are presented as frequencies and proportions. For the analysis of brain injury outcomes, continuous variables were compared using the two-sample Student’s *t*-test, whereas categorical variables were compared using the chi-square test of Fisher’s exact test. Case–control matching was applied for the comparison between the two groups. Statistical significance was set at *p* < 0.05. All statistical analyses were performed using MedCalc^®^ Statistical Software version 22.016 (MedCalc Software Ltd., Ostend, Belgium; https://www.medcalc.org, accessed on 24 April 2024, 2023).

## 3. Results

Of the 60 patients, all completed the preoperative MMSE, qEEG, and serum injury marker sampling, as well as intraoperative serum injury marker sampling and rSO_2_ monitoring. Fifty-eight patients underwent MMSE and qEEG evaluations on POD 7. Two patients did not complete the POD 7 evaluations due to unstable clinical conditions. Fifty-three patients underwent MMSE and EEG evaluations on POD 60. Seven patients did not complete the POD 60 evaluations: One patient expired on POD 16, and six patients refused the POD 60 follow-up. There were no significant differences in the preoperative characteristics between the two groups (Table 1). The operative data are listed in Table 2. There were no cases of coronary artery bypass grafting in the HTK group (*p* = 0.0001), and the number of patients who underwent valve surgery was significantly higher in the HTK group than in the Plegisol group (*p* = 0.010). Both the CPB and ischemic time durations were significantly longer in the HTK group than in the Plegisol group (*p* = 0.0009, and 0.031, respectively). In addition, the reduction in regional cerebral oxygen saturation (rSO_2_) from baseline by >20% was significantly higher in the HTK group than in the Plegisol group (*p* = 0.014).

**Figure 2:**
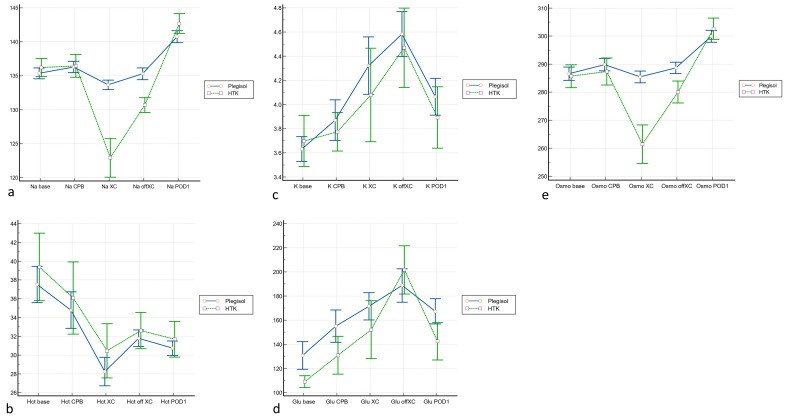
Intraoperative and postoperative sodium, potassium, glucose, and hematocrit levels were examined: (**a**) Regarding sodium levels, the HTK group exhibited significantly lower levels than the Plegisol group in both post-XC (*p* < 0.0001) and off-XC (*p* < 0.0001) periods. However, on POD 1, the sodium level was significantly higher in the HTK group than in the Plegisol group. (**b**) Regarding potassium levels, no significant differences were observed between the two groups at any stage. (**c**) No significant differences were noted in the hematocrit levels between the two groups across all stages. (**d**) Regarding glucose levels, only the baseline glucose level was lower in the HTK group than in the Plegisol group (*p* = 0.05), although no significant differences were found in the other stages. (**e**) Osmolality exhibited similar alterations to the sodium levels. On POD 1, the osmolality in the HTK group did not demonstrate a significant elevation compared with the Plegisol group. Abbreviations: base, baseline; CPB, cardiopulmonary bypass; Glu, glucose; Hct, hematocrit; POD 1, postoperative day 1; Osmo, osmolality; XC, cross-clamp.

The post-XC and off-XC sodium levels in the HTK group were significantly lower (*p* < 0.0001 and <0.0001, respectively, Figure 2a). After releasing the cross-clamp, the sodium level in the HTK group rapidly increased, which was higher than that in the Plegisol group on POD 1 (*p* = 0.034, Table S1). The osmolality exhibited parallel alterations to the sodium levels; however, on POD1, the osmolality in the HTK group did not demonstrate a significant elevation compared with that in the Plegisol group (Figure 2e). Furthermore, potassium, glucose, and Hct levels were not significantly different between the two groups (Figure 2b–d).

**Figure 3:**
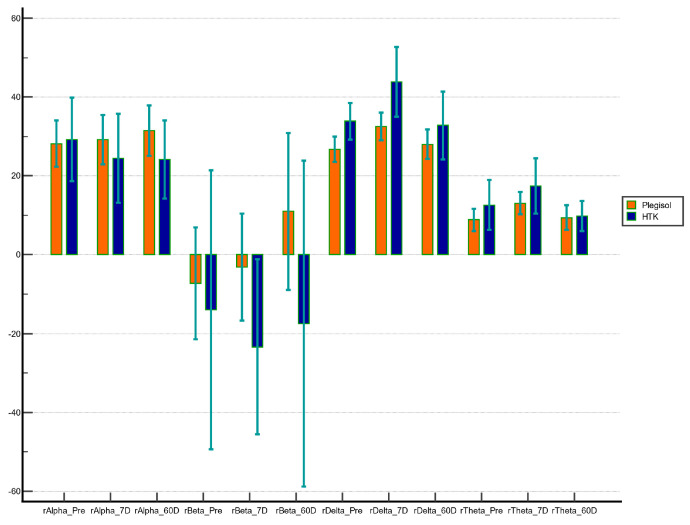
Relative alpha, beta, delta, and theta power variations in preoperative, postoperative day 7, and postoperative day 60. Error bars: 95% confidence interval of mean. Abbreviations: 7D, postoperative day 7; 60D, postoperative day 60; rAlpha, relative alpha power; rBeta, relative beta power; rDelta, relative delta power; rTheta, relative theta power.

The MMSE scores were not significantly different between the two groups, either preoperatively on POD 7 or POD 60 (Table 3). Regarding brain injury markers, the changes in S100β and Tau protein levels after CPB were not significantly different between the two groups. Notably, the NSE level changes after CPB were significantly higher in the HTK group than those in the Plegisol group. Moreover, the qEEG analysis showed a significantly higher relative delta power in the HTK group than that in the Plegisol group on POD 7 (*p* = 0.018) but not on POD 60 (*p* = 0.113). The difference in quantitative data on qEEG waves, including alpha, beta, and theta powers, were not statistically significant between the two groups on POD 7 (*p* = 0.558, 0.337, and 0.513, respectively) or POD 60 (*p* = 0.917, 0.351, and 0.134, respectively) (Figure 3).

To compare the neurological outcomes between the HTK and Plegisol groups without confounding factors, we performed a case–control matching analysis based on age (within 10 years), sex (exact match), CPB duration (within 60 min), and surgical procedure (exact match). After matching, no significant differences in preoperative and intraoperative characteristics were observed between the two groups (Table S2). Regarding neurological outcomes, the MMSE scores were not significantly different between the two groups (Table 4). The brain injury markers showed no significant differences in the S100β, NSE, or Tau protein level after CPB between the two groups. However, the qEEG analysis on POD 7 revealed a significantly higher relative delta power in the HTK group than in the Plegisol group (*p* = 0.017). Although there was no statistical significance on POD 60, the relative delta power showed a trend of difference between the two groups (*p* = 0.092).

## 4. Discussion

In this study, we evaluated the changes in sodium levels during CPB and the ischemic stage between the HTK and Plegisol groups. Acute hyponatremia was observed in the HTK group after cross-clamping, with the sodium levels showing a significant difference between the HTK and Plegisol groups. Moreover, the rapid correction of hyponatremia was observed after the release of the cross-clamp, resulting in even higher sodium levels in the HTK group than in the Plegisol group on POD 1. For the brain injury markers, the NSE level change after CPB was significantly higher in the HTK group than in the Plegisol group, but the difference was insignificant in the case–control matching analysis. Although the MMSE scores on POD 7 and POD 60 were not significantly different between the two groups, the qEEG analysis revealed a significantly higher relative delta power in the early postoperative period, followed by a trend of differences on POD 60. Although the biological changes were not reflected as clinical symptoms, the neurological impact of HTK infusion requires further investigation among adult cardiac surgical patients.

Previous studies have shown that the use of HTK in pediatric cardiac surgeries can cause acute hyponatremia, which can lead to a higher risk of postoperative seizures [9,12]. Although the rapid correction of HTK-induced hyponatremia in pediatric patients has been considered harmful [16], right atrial effluent scavenging has been approved in this age group to prevent sodium level fluctuations and further development of postoperative seizures [17]. In contrast, although the use of HTK in adult cardiac surgeries also results in acute hyponatremia, there is no increased risk of postoperative seizures or other brain insults. Therefore, rapid hyponatremia correction with hypertonic saline during CPB is considered unnecessary [13,18]. However, another study revealed that patients receiving HTK cardioplegia during coronary bypass surgery had a higher incidence of postoperative delirium [19]. There are limited studies that evaluated the relationship between HTK, hyponatremia, rapid correction, right atrial effluent scavenging, and brain insults in adult cardiac surgeries; therefore, the management strategy for HTK-related hyponatremia remains controversial. According to previous studies [20,21], the rapid correction of acute hyponatremia within 24–48 h after its onset does not produce long-term neurological sequelae, including osmotic demyelination. In our institution, we routinely use ultrafiltration with normal saline to correct acute hyponatremia induced by HTK cardioplegia. In our study, no patient presented with postoperative seizures or symptoms of demyelination.

Regarding neurological outcomes, the MMSE scores on POD 7 and POD 60 were not significantly different between the two groups. The brain injury marker NSE showed a significant increase in the HTK group, which may be attributed to the longer CPB and ischemic times in this group. After case–control matching for CPB duration and surgical procedure, no significant difference in NSE levels was observed between the two groups. However, the qEEG analysis showed a significantly higher relative delta power on POD 7 in the HTK group than in the Plegisol group, before and after matching. On POD 60, no significant difference in relative delta power was found between the two groups. As hyponatremia-induced and rapid correction-induced EEG changes manifest as diffuse slow activity, particularly delta waves [11,22,23], it becomes challenging to discern whether the heightened relative delta power observed in the HTK group can be attributed to hyponatremia, rapid correction, or a combination of both. Nevertheless, the difference in relative delta power between the two groups became insignificant on POD 60, indicating that the qEEG changes are transient and may not lead to long-term sequelae.

In this study, no patient in the HTK group underwent isolated CABG. Surgeons preferred using HTK for valve surgeries, which led to significant differences in procedures and CPB or ischemia durations between the two groups before case–control matching. The higher NSE levels before matching in the HTK group were likely because of these differences. Therefore, the differences observed after matching are likely attributable to the comparison between HTK and Plegisol.

To the best of our knowledge, this study is the first to investigate preoperative and postoperative MMSE scores, brain injury markers, and qEEG findings in adult patients undergoing cardiac surgery with HTK or Plegisol cardioplegia. According to our findings, we hypothesize that intraoperative hyponatremia induced by HTK solution and its prompt correction with normal saline during adult cardiac surgeries may have short-term neurological implications, yet it may not adversely affect the long-term neurological outcomes of patients. However, further large-scale prospective randomized studies are required to determine the necessity of rapid correction of hyponatremia intraoperatively.

### Limitations

This study has several limitations that should be considered. First, the present study was performed using a single institution and had a prospective cohort study design with retrospective data collection, which may introduce selection bias. Second, the sample size was limited, particularly in the HTK group, which makes propensity score matching between the two groups unfeasible. Third, HTK or Plegisol cardioplegia was selected by the surgeons based on their subjective preference rather than being selected randomly, thereby introducing potential biases related to selection and experience. Fourth, detailed brain imaging, such as MRI, was not conducted, indicating that subtle strokes may have gone undetected. Fifth, because no patient in the HTK group underwent isolated CABG, our study results cannot be directly applied to CABG procedures. Consequently, whether the use of HTK or Plegisol would impact the neurological outcomes of patients with CABG could not be determined. Finally, as previously reported [14], the rate of discarded or non-qualified qEEG datasets was relatively high, indicating that the qEEG results may not accurately reflect the findings in all patients.

## 5. Conclusions

During CPB, acute hyponatremia was observed in the HTK group, which significantly differed from that observed in the Plegisol group. Although there were no statistically significant differences in MMSE scores on POD 7 and POD 60, qEEG analysis indicated a notable increase in delta power during the early postoperative period, although this difference was not observed on POD 60. Further studies should be conducted to compare the effects of correction and non-correction strategies for HTK-induced hyponatremia in adult patients to determine the necessity of rapid correction.

## Figures and Tables

**Table 1:** Baseline characteristics and preoperative data of the patients in the HTK and Plegisol groups.

Variable	HTK (*n* = 13)	Plegisol (*n* = 47)	*p*
Age (years)	58.0 (55.3–65.3)	61.0 (50.0–66.8)	0.507
Sex, male	10 (76.9)	35 (74.5)	0.857
Hypertension	6 (46.2)	29 (61.7)	0.318
DM	2 (15.4)	21 (44.7)	0.105
Dialysis	1 (7.7)	4 (8.5)	1.000
CCr (mL/min)	110.3 (87.5–122.1)	69.5 (50.2–89.9)	0.070
COPD	0 (0.0)	2 (4.3)	1.000
Old CVA	2 (15.4)	3 (6.4)	0.294
PAOD	1 (7.7)	8 (17.0)	0.668
Carotid stenosis ^a^	1 (7.7)	8 (17.0)	0.668
NYHA Fc (II/III/IV)	4/5/4 (30.8/38.5/30.8)	10/24/13 (21.3/51.1/27.7)	0.683
LVEF (%)	64.0 (58.0–72.6)	61.2 (51.4–68.7)	0.401
PASP (mmHg)	31.0 (25.0–48.8)	30.0 (25.0–47.3)	0.346
Preoperative Hb (g/dL)	13.7 (11.7–15.1)	12.6 (11.1–14.3)	0.356
NTproBNP (pg/mL)	297.0 (146.5–1185.0)	627.0 (142.0–1898.3)	0.449
AST (IU/L)	25.0 (18.0–28.5)	24.0 (20.0–33.0)	0.756
ALT (IU/L)	18.0 (13.5–44.3)	20.0 (15.3–27.0)	0.851

Values are number (%) or median (25th–75th percentile). ^a^ Carotid stenosis is defined as carotid artery stenosis >50% documented by carotid duplex, computed tomography, or angiography. Abbreviations: AST, aspartate aminotransferase; ALT, alanine aminotransferase; CCr, creatinine clearance rate; CVA, cerebrovascular events; DM, diabetes mellitus; Hb, hemoglobin; LVEF, left ventricular ejection fraction; NTproBNP, N terminal pro B type natriuretic peptide; NYHA Fc, New York Heart Association Classification functional class; PAOD, peripheral arterial occlusive disease; PASP, pulmonary artery systolic pressure.

**Table 2:** Intraoperative data and cerebral regional tissue oxygen saturation.

Variable	HTK (*n* = 13)	Plegisol (*n* = 47)	*p*
Isolated CABG	0 (0.0)	27 (57.4)	0.0001 *
Valve	9 (69.2)	14 (29.8)	0.010 *
Valve + CABG	2 (15.4)	4 (8.5)	0.601
Aorta	2 (15.4)	2 (4.3)	0.201
CPB CI (mL/min)	2.89 (2.69–3.08)	2.98 (2.82–3.08)	0.155
CPB duration (min)	213.0 (168.3–285.5)	167.0 (127.3–206.8)	0.0009 *
Ischemia time (min)	156.0 (110.8–192.3)	117.0 (95.8–144.3)	0.031
Hb level (g/dL)	10.5 (8.7–11.3)	9.0 (8.0–10.7)	0.154
Transfusion unit (U)	4.0 (0.0–6.0)	4.0 (0.0–6.0)	0.803
Baseline rSO_2_ (%)	67.0 (51.3–68.3)	58.5 (50.0–66.5)	0.414
Mean rSO_2_ (%)	60.0 (58.3–63.8)	61.0 (54.6–70.9)	0.632
Lowest rSO_2_ (%)	43.0 (37.4–46.8)	45.5 (38.4–53.4)	0.242
AUC of rSO_2_ < 40% (%min)	0.0 (0.0–24.6)	0.0 (0.0–13.9)	0.838
Reduction in rSO_2_ > 20% (%min)	59.5 (12.8–150.4)	1.0 (0.0–24.6)	0.014 *

Values are number (%) or median (25th–75th percentile). * Significant difference. Abbreviations: AUC, area under the curve; CABG, coronary artery bypass grafting; CI, cardiac index; CPB, cardiopulmonary bypass; Hb, hemoglobin; rSO_2_, cerebral regional tissue oxygen saturation.

**Table 3:** Postoperative neurological outcomes between the HTK and Plegisol groups.

	HTK (*n* = 13)	Plegisol (*n* = 47)	*p*
MMSE pre	29.0 (26.8–29.3)	27.0 (26.0–29.0)	0.758
MMSE D7	28.0 (27.3–29.0)	27.0 (26.0–28.0)	0.062
MMSE D60	28.0 (26.5–29.8)	28.0 (27.0–29.0)	0.290
S100β diff	157.1 (118.5–239.9)	107.7 (50.7–184.3)	0.302
NSE diff	8.3 (7.8–12.4)	5.3 (3.9–10.9)	0.043 *
Tau diff	258.1 (184.5–393.7)	347.7 (205.5–741.2)	0.139
rAlpha pre	27.8 (18.5–41.0)	34.2 (20.6–44.4)	0.425
rAlpha 7D	28.1 (16.5–36.6)	33.1 (22.9–43.1)	0.558
rAlpha 60D	25.8 (17.3–43.7)	26.6 (20.3–45.3)	0.917
rBeta pre	−23.4 (−28.2–−7.2)	−2.3 (−33.3–31.6)	0.133
rBeta 7D	−9.8 (−33.8–15.4)	−1.1 (−19.9–27.2)	0.337
rBeta 60D	2.6 (−43.3–27.8)	12.9 (−17.3–60.3)	0.351
rDelta pre	32.2 (29.0–33.7)	29.2 (22.5–33.2)	0.173
rDelta 7D	43.1 (36.4–46.9)	33.2 (26.5–38.1)	0.018 *
rDelta 60D	31.4 (28.3–37.5)	26.9 (22.8–32.0)	0.113
rTheta pre	10.0 (7.1–15.6)	10.9 (7.0–17.3)	0.938
rTheta 7D	18.4 (11.5–23.2)	14.1 (9.2–21.1)	0.513
rTheta 60D	12.0 (6.9–27.0)	8.3 (4.0–15.6)	0.134

Values are median (25th–75th percentile). * Significant difference. Abbreviations: 7D, postoperative day 7; 60D, postoperative day 60; diff, difference between preoperative and off-pump brain injury marker level; MMSE, Mini-Mental State Examination; NSE, neuron-specific enolase; pre, preoperative; rAlpha, relative alpha power; rBeta, relative beta power; rDelta, relative delta power; rTheta, relative theta power.

**Table 4:** Postoperative neurological outcomes in the HTK and Plegisol groups after case–control matching.

	Post-Case–Control Matching		
	HTK (*n* = 10)	Plegisol (*n* = 10)	*p*
MMSE pre	29.0 (27.0–30.0)	27.0 (26.0–29.0)	0.771
MMSE D7	28.0 (26.8–29.0)	27.5 (25.0–28.0)	0.157
MMSE D60	28.5 (28.0–30.0)	29.0 (27.8–29.0)	0.619
S100β diff	134.1 (101.8–191.0)	46.1 (27.6–78.2)	0.495
NSE diff	8.3 (7.0–9.1)	9.2 (4.9–11.8)	0.878
Tau diff	244.8 (171.4–388.2)	407.2 (262.5–998.6)	0.071
rAlpha pre	25.9 (18.5–33.7)	32.5 (21.9–45.1)	0.401
rAlpha 7D	26.3 (13.0–34.1)	26.7 (23.9–47.5)	0.603
rAlpha 60D	25.8 (18.2–51.9)	19.0 (15.9–21.9)	0.117
rBeta pre	−23.2 (−36.7–−10.4)	−2.8 (−24.8–60.9)	0.181
rBeta 7D	−15.0 (−35.5–17.4)	17.8 (−30.1–38.5)	0.308
rBeta 60D	−7.2 (−45.3–18.8)	7.1 (−23.9–14.4)	0.947
rDelta pre	31.4 (29.1–33.7)	31.6 (25.4–33.2)	0.290
rDelta 7D	44.2 (38.9–48.2)	36.8 (23.7–39.2)	0.017 *
rDelta 60D	31.4 (28.8–36.7)	26.9 (23.5–28.4)	0.092
rTheta pre	11.2 (6.7–19.3)	16.1 (4.3–22.7)	0.799
rTheta 7D	18.8 (13.9–25.1)	17.3 (8.9–21.4)	0.569
rTheta 60D	12.9 (8.6–27.9)	5.0 (1.3–9.1)	0.075

Values are median (25th–75th percentile). * Significant difference. Abbreviations: 7D, postoperative day 7; 60D, postoperative day 60; diff, difference between preoperative and off-pump brain injury marker level; MMSE, Mini-Mental State Examination; NSE, neuron-specific enolase; pre, preoperative; rAlpha, relative alpha power; rBeta, relative beta power; rDelta, relative delta power; rTheta, relative theta power.

## Data Availability

The raw data supporting the conclusions of this article will be made available by the authors upon request.

## Supplementary Materials

The following supporting information can be downloaded at: https://www.mdpi.com/article/10.3390/medicina60060995/s1, Table S1: The intraoperative and postoperative sodium, potassium, glucose, and hematocrit; Table S2: Preoperative and intraoperative data between the HTK and Plegisol groups after case–control matching.
