# Supplementary material for: The Potential Neurological Impact of Intraoperative Hyponatremia Using Histidine–Tryptophan–Ketoglutarate Cardioplegia Infusion in Adult Cardiac Surgery
Source: Medicina (Kaunas). 2024 Jun 18;60(6):995. doi: 10.3390/medicina60060995 (PMC11205320; doi:10.3390/medicina60060995)
Supplement: Supplementary file 1 [file medicina-60-00995-s001.zip › medicina-3005382-supplementary.pdf]

Supplementary Table S1. The intraoperative and postoperative sodium, potassium, glucose and hematocrit

| | HTK (n=3) | Plegisol (n=47) | |
|---|---|---|---|
| **Na base** | 136.23 ± 2.20 | 135.36 ± 2.72 | 0.294 |
| **Na CPB** | 136.46 ± 2.79 | 136.30 ± 2.87 | 0.855 |
| **Na XC** | 122.92 ± 4.72 | 133.66 ± 2.43 | <0.0001* |
| **Na off-XC** | 130.69 ± 1.80 | 135.30 ± 2.96 | <0.0001* |
| **Na POD1** | 142.69 ± 2.39 | 140.74 ± 2.99 | 0.034 |
| **K base** | 3.70 ± 0.35 | 3.63 ± 0.35 | 0.541 |
| **K CPB** | 3.77 ± 0.27 | 3.87 ± 0.57 | 0.560 |
| **K XC** | 4.08 ± 0.64 | 4.32 ± 0.81 | 0.321 |
| **K off-XC** | 4.47 ± 0.54 | 4.58 ± 0.63 | 0.557 |
| **K POD1** | 3.89 ± 0.42 | 4.06 ± 0.52 | 0.278 |
| **Glu base** | 109.15 ± 8.13 | 130.83 ± 38.57 | 0.050 |
| **Glu CPB** | 131.00 ± 25.88 | 154.96 ± 45.61 | 0.075 |
| **Glu XC** | 152.15 ± 39.63 | 171.47 ± 38.73 | 0.118 |
| **Glu off-XC** | 201.62 ± 33.09 | 188.72 ± 46.96 | 0.358 |
| **Glu POD1** | 142.62 ± 25.57 | 167.36 ± 35.75 | 0.023 |
| **Osmo base** | 285.69 ± 6.77 | 286.60 ± 8.16 | 0.715 |

| | | | |
|---|---|---|---|
| **Osmo CPB** | 287.37 ± 7.98 | 289.81 ± 7.55 | 0.312 |
| **Osmo XC** | 261.47 ± 11.41 | 285.45 ± 7.15 | <0.0001* |
| **Osmo off-XC** | 280.05 ± 6.46 | 288.69 ± 6.84 | 0.0001* |
| **Osmo POD1** | 302.67 ± 6.29 | 299.93 ± 7.24 | 0.220 |
| **Hct base** | 39.38 ± 5.97 | 37.51 ± 6.57 | 0.357 |
| **Hct CPB** | 36.08 ± 6.36 | 34.79 ± 6.66 | 0.535 |
| **Hct XC** | 30.46 ± 4.75 | 28.26 ± 5.15 | 0.170 |
| **Hct off XC** | 32.62 ± 3.20 | 31.79 ± 3.01 | 0.390 |
| **Hct POD1** | 31.68 ± 3.13 | 30.73 ± 2.64 | 0.277 |

Values are mean ± SD.
*Significant difference

Abbreviations: base, baseline; CPB, cardiopulmonary bypass; Glu, glucose; Hct, hematocrit;

Osmo, osmolality; POD1, postoperative D1, XC, cross-clamp

Supplementary Table S2. Preoperative and intraoperative data between the HTK and Plegisol groups after case—control matching

| **Variable** | **HTK (n = 10)** | **Plegisol (n = 10)** | ***p*** |
|---|---|---|---|
| **Age (years)** | 59.0(57.0-66.0) | 62.5(50.0-66.0) | 0.930 |
| **Sex, male** | 8(80) | 8(80) | 1.000 |
| **DM** | 2(20) | 2 (20) | 1.000 |
| **CCr (ml/min)** | 111.1(88.9-122.0) | 80.8(50.5-99.8) | 0.410 |
| **Preoperative Hb (g/dL)** | 14.1(11.7-15.0) | 13.9(11.0-15.1) | 0.664 |
| **Isolated CABG** | 0(0) | 0(0) | 1.000 |
| **Valve** | 7(70) | 8(80) | 0.614 |
| **CPB CI (L/min)** | 2.9(2.7-3.1) | 2.9(2.7-3.0) | 0.875 |
| **CPB duration (min)** | 184.5(163.0-258.0) | 203.5(181.0-233.0) | 0.928 |
| **Ischemia time (min)** | 118.5(110.0-173.0) | 131.0(105.0-155.0) | 0.986 |
| **Hb level (g/dL)** | 10.7(8.7-11.7) | 10.3(8.4-10.9) | 0.512 |
| **Transfusion unit (U)** | 2.0(0.0-6.0) | 2.0(0.0-6.0) | 0.674 |
| **baseline rSO2 (%)** | 64.7(47.5-68.0) | 55.5(47.0-63.5) | 0.473 |
| **mean rSO2 (%)** | 61.5(58.5-64.5) | 58.3(54.5-68.0) | 0.611 |
| **lowest rSO2 (%)** | 43.2(37.0-52.0) | 38.7(37.0-49.5) | 0.936 |
| **AUC of rSO2 < 40% (%min)** | 0.0 (0.0-10.5) | 8.25(0.0-23.5) | 0.844 |
| **reduction of rSO2 > 20% (%min)** | 19.5(12.0-81.5) | 0.5(0.0-397.0) | 0.949 |

Values are number (%) or median ($25^{th}$–$75^{th}$ percentile).
*Significant difference

Abbreviations: AUC, area-under-curve; CABG, coronary artery bypass grafting; CCr, creatinine clearance rate; CI, cardiac index; CPB, cardiopulmonary bypass; DM, diabetes mellitus; Hb, hemoglobin; rSO2, cerebral regional tissue oxygen saturation.
